# Social reappraisal of emotions is linked with the social presence effect in the default mode network

**DOI:** 10.3389/fpsyt.2023.1128916

**Published:** 2023-03-23

**Authors:** Xiyao Xie, Teresa Bertram, Saša Zorjan, Marina Horvat, Christian Sorg, Satja Mulej Bratec

**Affiliations:** ^1^Department of Neuroradiology, Klinikum rechts der Isar, Technical University of Munich, Munich, Germany; ^2^Department of Psychiatry and Psychotherapy, Klinikum rechts der Isar, Technical University of Munich, Munich, Germany; ^3^Department of Psychology, Faculty of Arts, University of Maribor, Maribor, Slovenia

**Keywords:** social reappraisal, social support, social emotion regulation, social presence, default mode network, interpersonal trust, anterior cingulate

## Abstract

**Introduction:**

Social reappraisal, during which one person deliberately tries to regulate another’s emotions, is a powerful cognitive form of social emotion regulation, crucial for both daily life and psychotherapy. The neural underpinnings of social reappraisal include activity in the default mode network (DMN), but it is unclear how social processes influence the DMN and thereby social reappraisal functioning. We tested whether the mere presence of a supportive social regulator had an effect on the DMN during rest, and whether this effect in the DMN was linked with social reappraisal-related neural activations and effectiveness during negative emotions.

**Methods:**

A two-part fMRI experiment was performed, with a psychotherapist as the social regulator, involving two resting state (social, non-social) and two task-related (social reappraisal, social no-reappraisal) conditions.

**Results:**

The psychotherapist’s presence enhanced intrinsic functional connectivity of the dorsal anterior cingulate (dACC) within the anterior medial DMN, with the effect positively related to participants’ trust in psychotherapists. Secondly, the social presence-induced change in the dACC was related with (a) the social reappraisal-related activation in the bilateral dorsomedial/dorsolateral prefrontal cortex and the right temporoparietal junction and (b) social reappraisal success, with the latter relationship moderated by trust in psychotherapists.

**Conclusion:**

Results demonstrate that a psychotherapist’s supportive presence can change anterior medial DMN’s intrinsic connectivity even in the absence of stimuli and that this DMN change during rest is linked with social reappraisal functioning during negative emotions. Data suggest that trust-dependent social presence effects on DMN states are relevant for social reappraisal—an idea important for daily-life and psychotherapy-related emotion regulation.

## 1. Introduction

Social reappraisal is a powerful cognitive form of social emotion regulation, important in both daily life and psychotherapy (1, 2). It represents the process by which one person deliberately tries to change emotions of another using a cognitive approach, such as suggesting a reinterpretation of an emotional stimulus or situation (2). We previously showed that social reappraisal of negative stimuli by a psychotherapist is supported by the brain’s default mode network (DMN) (3), primarily comprising the medial prefrontal cortex (MPFC), the posterior cingulate (PCC) and precuneus, the lateral parietal cortex, and the temporoparietal junction (TPJ) (4, 5). However, a crucial next step would be to determine which DMN processes might influence the neural activity and effectiveness of social reappraisal, especially considering the DMN’s role as “an active and dynamic ‘sense-making’ network integrating incoming extrinsic information with prior intrinsic information to form rich, context-dependent models of situations as they unfold over time” (6). We thus asked, firstly, whether the simple presence of a supportive social regulator will impact the DMN state as realized *during rest,* and secondly, whether this effect will be associated with both social reappraisal effectiveness and brain activations *during negative emotions.*

**Figure 1:**
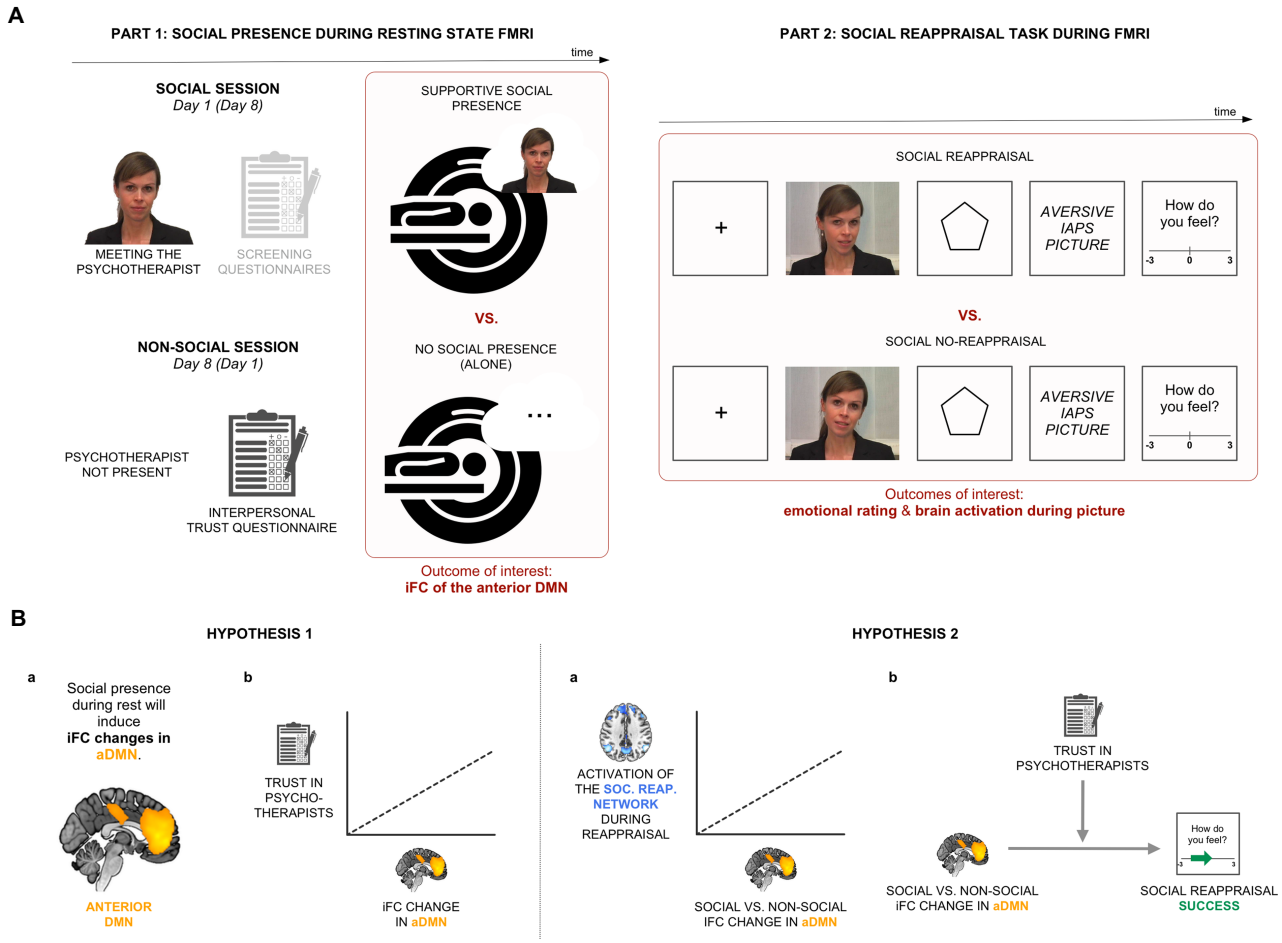
Experimental design and hypotheses. **(A)** In Part 1 of the experiment, we recorded participants’ brain activity in two resting-state conditions, alone and in the condition of perceived supportive presence of a psychotherapist. Part 2 involved a social reappraisal task with aversive pictures as negative stimuli. **(B)** Hypothesis 1: We hypothesized that iFC of the anterior medial default mode network (aDMN) will be sensitive to the psychotherapist’s presence (Hypothesis 1a) and that the effect will be related to general trust in psychotherapists (Hypothesis 1b). Hypothesis 2: We expected a positive relationship between the social presence-induced iFC change during rest and brain activations within the social reappraisal activation network (Hypothesis 2a). We further hypothesized a positive relationship between the social presence effect in the aDMN and social reappraisal success, moderated by trust in psychotherapists (Hypothesis 2b).

Intrinsic brain networks are based on coherent ongoing (i.e., intrinsic) activity and are thereby detectable at various resting state conditions, such as resting with eyes open or closed. The networks are commonly investigated by resting-state blood oxygenation level dependent (BOLD) functional MRI (fMRI) *via* correlated BOLD fluctuations in the infra-slow frequency spectrum (<0.1 Hz), referred to as intrinsic functional connectivity (iFC) (7–9). To detect iFC changes in the DMN during rest, we recorded participants’ brain activity in two resting-state conditions—once alone and once in the condition of perceived supportive presence of a psychotherapist—on different days (see Figure 1A-left). Despite being highly stable between and within individuals (10, 11), iFC is sensitive to specific short-term experiences (12), and exhibits changes across time and mental states (10, 13). DMN connectivity, specifically, changes in response to external context, such as spoken narratives (14). However, no study to date has investigated whether the DMN’s iFC might be changed by the supportive presence of a trustworthy other. The powerful positive impact the presence of a trustworthy conspecific can have on our physical and emotional state (15–17) led us to expect that supportive social presence of a psychotherapist will impact the DMN’s iFC, more specifically iFC of the anterior medial DMN (aDMN) (Hypothesis 1a, see Figure 1B-left). The human aDMN covers the MPFC, a region signaling safety and social proximity (16, 18); the orbitofrontal cortex (OFC), involved in social evaluation and encoding of social rewards (19–21); and the dorsal anterior cingulate cortex (dACC), a region responding to social exclusion and lack of social support (22, 23). Furthermore, a study found that voxels in the dACC and OFC showed higher response magnitudes while alone, compared to holding a friend’s hand, even during the resting periods of a shock experiment (24).

A psychotherapist was chosen as the partner for supportive social presence (and social reappraisal), as a prototypical role model for coping with negative emotions; psychotherapists also perceive themselves as highly able to cope with negative emotional feelings (25, 26). Nevertheless, people vary in their beliefs of psychotherapists’ trustworthiness and the level of general trust in psychotherapists might relate to the level of positive influence a specific psychotherapist will have on an individual (27, 28). As DMN activity and connectivity have been shown to change in response to prior beliefs and schemas (6, 29, 30), we hypothesized that higher general trust in psychotherapists might relate to a stronger social presence-induced iFC change in the aDMN (Hypothesis 1b; see Figure 1B-left).

DMN, as a brain system, is seen as the integrator of extrinsic inputs and intrinsic memories and beliefs, which thus supports and promotes social interactions (6). Focusing on a specific social interaction, we previously found that DMN activations support social reappraisal (3). Here, we were interested in whether and how changes within the DMN (e.g., due to supportive social presence) might influence social reappraisal functioning—both DMN activations during social reappraisal and social reappraisal effectiveness. A study found that the reappraisal of negative images was strongest when initiated by a close friend compared to a stranger or the participants themselves, implying a positive relationship between the processes of supportive social presence and social reappraisal (31). In terms of neural activations, we thus hypothesized that changes in aDMN connectivity due to supportive presence of the psychotherapist will positively relate with social reappraisal-related brain activations during negative emotions (i.e., the “social reappraisal activation network”) (Hypothesis 2a, see Figure 1B-left). With regard to the behavioral outcome, we expected that the social presence effect during rest might also relate to social reappraisal success, but, in line with the social presence effect, depending on the person’s preconceived trust in psychotherapists (Hypothesis 2b; see Figure 1B-right).

## 2. Materials and methods

### 2.1. Participants

Twenty-two healthy female participants (aged 24.95 ± 2.30) completed the study. Only females were recruited due to previously reported gender differences regarding the neural basis of emotional processing (32, 33) and emotion regulation (34, 35). The decision was made as a compromise between the signal-to-noise ratio (related to sample homogeneity) and generalizability of our findings. Participants reported no history of mental or neurological disorders, no current use of psychoactive medications, used German as the dominant language, and had normal or corrected-to-normal vision. All participants were fMRI compatible (no metal on or in the body, not pregnant, not claustrophobic), provided written informed consent and received monetary compensation for their participation. The study was approved by the local ethics committee of the Klinikum rechts der Isar at the Technical University of Munich and carried out in accordance with the Declaration of Helsinki.

### 2.2. Experimental design and procedure

The study consisted of an experiment with two parts (see Figure 1A). Both Part 1 (social presence during resting-state functional MRI [rs-fMRI]; Figure 1A-left) and Part 2 (social reappraisal task during fMRI; Figure 1A-right) followed a within-subjects design with two conditions each: social vs. non-social and social reappraisal vs. social no-reappraisal, respectively. To be able to explore the connection between social presence and social reappraisal by the same psychotherapist, Part 2 was conducted after the resting state scan of the social session in Part 1.

#### 2.2.1. Part 1: Social presence during rs-fMRI

In the social condition, participants were told that they will be accompanied by a female psychotherapist during the whole experiment. They were introduced to her (a fully trained and practicing in-house psychotherapist) for the first time before scanning: she introduced herself and told participants that she will be in the adjacent room, able to see them, and talk to them *via* a camera-microphone system during the experiment. In the non-social condition, participants were informed that they will perform the experiment alone. The two conditions were conducted 1 week apart, with the condition order counterbalanced across participants.

A standard procedure of rs-fMRI scanning was used, lasting 8 min. Participants were told to lie still with their eyes closed and not fall asleep. Except of the in-person conversation with the psychotherapist before scanning, participants had no further contact with her during Part 1 of the experiment. A post-scanning interview verified that all participants both believed that she was present throughout scanning and stayed awake during the 8-min-long scan.

#### 2.2.2. Part 2: Social reappraisal task during fMRI

In the social reappraisal task, aversive pictures (from the International Affective Picture System, IAPS) were presented to induce negative emotions (see Figure 1A-right). The picture list can be found in Supplementary material. Shortly before each stimulus, the psychotherapist either actively regulated participants’ emotional responses to the pictures (condition of “social reappraisal”) or passively instructed participants to view the pictures (condition of “social no-reappraisal”), as in a previous study (3). Participants believed that the psychotherapist was instructing them live while sitting in the adjacent room throughout the experiment. However, pre-recorded videos of the psychotherapist were used, a different one on every trial, with her wearing the same hairstyle and clothes as when participants met her in person immediately prior to scanning. This was chosen for both practical and experimental reasons to ensure that stimuli were standardized across participants. In the social no-reappraisal condition, she used statements such as “Please look at the picture,” while in the social reappraisal condition, she asked participants to follow her reappraisals of the negative pictures by using statements such as “Remember, the pictures are not related to you,” “Keep in mind that the pictures are staged,” or “This is an experiment, not reality.” The list of sentences can be found in Supplementary material. Participants confirmed in a post-scanning interview that they engaged in the emotional task and followed the psychotherapist’s guidance during the two conditions of social reappraisal and social no-reappraisal.

The social reappraisal and social no-reappraisal conditions each involved 80 trials, with the negative pictures presented on half of the trials, with only the “picture present” trials having been analyzed for the purpose of this publication. Every trial involved a unique video clip of the psychotherapist and a unique negative picture. With regard to the trial structure, the fixation cross (1 s) and the psychotherapist’s video instructions (6 s) were followed by the anticipation phase (6 s) and aversive picture presentation (6 s) (Figure 1A-right). At the end of each trial, participants rated their emotional feelings on a scale from −3 (very negative) to 3 (very positive), which was set to 0 on each trial. Participants responded with button presses, such that each press moved the cursor by one place in the desired direction. To evaluate the effectiveness of social reappraisal, social reappraisal success was calculated as the difference in emotional ratings between the social reappraisal and social no-reappraisal conditions.

#### 2.2.3. Participants’ trust In psychotherapists

To check participants’ general belief in psychotherapists, we administered a German interpersonal trust questionnaire (“Inventar zur Erfassung interpersonellen Vertrauens”) (36) and analyzed the subscale “Trust in Psychotherapists,” which includes the following 3 items: “Psychotherapists have the ability to help people find their way out of a mental crisis,” “You can fully rely on the psychotherapists’ discretion,” and “Psychotherapists can offer great support in severe crises.” Each item is answered based on a 5-point Likert scale ranging from 1 (strongly disagree) to 5 (strongly agree). The range of participants’ trust in psychotherapists was 3–5 (mean = 4.35, SD = 0.53; for the distribution of values, see Supplementary Figure 2).

### 2.3. fMRI acquisition and preprocessing

MRI was performed on a 3 T MRI scanner (Verio, Siemens) using an 8-channel phased array head coil. T1-weighted structural images were acquired with the Magnetization-Prepared Rapid Acquisition Gradient Echo (MPRAGE) sequence (TE = 29.8 ms, TR = 2,300 ms, flip angle = 90°, acquisition matrix = 240 × 256, voxel size = 1 × 1 × 1 mm^3^). T2*-weighted functional images were acquired with Echo-Planar Imaging (EPI) pulse sequence (TE = 30 ms, TR = 2,000 ms, flip angle = 90°, acquisition matrix = 64 × 64, 35 slices, each 3 mm thick, with a gap of 0.6 mm, and in-plane resolution = 3 × 3 mm).

Data analysis was carried out with SPM12 and SPM8 (Wellcome Trust, London, United Kingdom; www.fil.ion.ucl.ac.uk/spm/). Preprocessing included the removal of the first two volumes of each condition, head motion correction, spatial normalization, and smoothing, with the additional step of slice-timing for task fMRI data. Using rigid body transformation, realignment of functional images to the first functional image of the first scanning session was performed. For normalization, the T1 image was segmented into gray and white matter and cerebrospinal fluid and transformed into the MNI system template. The normalization parameters were used to transform the functional images into the MNI space. Normalized functional images were smoothed with a Gaussian kernel (FWHM = 8 mm). While all 22 participants were used to answer hypotheses 1a, 1b, and 2b, 2 participants were excluded from task fMRI analysis for hypothesis 2a due to excessive movement (point-to-point translation >3 mm and/or rotation >3°).

### 2.4. Outcome measures and statistical hypothesis testing

#### 2.4.1. Testing hypothesis 1a: Rs-fMRI analysis

Preprocessed resting-state fMRI data of both social and non-social conditions were entered into a two-step principal component analysis (PCA), followed by a group-independent component analysis (ICA), as implemented in the GIFT toolbox,^1^ to decompose spatially independent components using the Infomax algorithm. First, PCA was done for each participant, which retained 100 principal components. The reduced data of each participant were then temporally concatenated to perform the second PCA on the group level. Seventy five independent components were estimated and depicted as spatial maps, together with 75 corresponding time series reflecting the dynamic activities of independent components. To obtain robust ICA results, 100 iterations of the ICA were carried out by using the ICASSO toolbox.^2^ The ICASSO index (ranging from 0 to 1) was evaluated to ensure the stability of estimated components. Finally, the group ICA results were back-reconstructed for each subject space by using the GICA3 back-reconstruction approach. Each back-reconstructed component consisted of a spatial intensity map reflecting component’s functional connectivity pattern across space, and an associated time-course reflecting component’s activity across time (37). Spatial intensity maps were used as surrogates of networks’ iFC and analyzed further.

High-model-order ICA approaches (75 components) yield independent components that are in accordance with known anatomical and functional segmentations (38, 39). In a high-model-order ICA approach, intrinsic brain networks, such as the DMN, are usually fragmented into several sub-networks (37). To select the independent components that reflected the aDMN and to objectively identify the entire DMN, we conducted multiple spatial regressions on 75 independent components’ spatial maps using templates of 4 intrinsic connectivity networks representing DMN sub-networks, which were defined by the same methodological approach in a prior study (i.e., the ICs 25, 68, 53, 50, presented in figure 4 in (37)). These templates were generated based on 603 healthy adolescents and adults and were made available online by the Medical Image Analysis Lab.^3^ For each DMN sub-network, the independent component with the largest correlation coefficient was chosen, resulting in: IC 7 as the aDMN (i.e., our network of interest), IC 35 as the anterior-lateral DMN, IC 40 as the posterior DMN, and IC 24 as the posterior superior DMN (see Supplementary Figure 1). Each of the selected ICs exhibited the highest spatial correlation with their corresponding template, and had a reliable ICASSO score (>0.95). To statistically evaluate the spatial map of our selected independent component IC 7, we carried out a voxel-wise one-sample *t*-test on participants’ back-reconstructed spatial intensity-maps for social and non-social conditions together, with a voxel-wise family-wise error (FWE) correction of *p* < 0.05.

To investigate the social presence effect in the aDMN (Hypothesis 1), the one-sample *t*-test result was used as a mask to restrict the search space, and a paired *t*-test was carried out for the aDMN component. Both contrasts (social > non-social and social < non-social) were examined. The resulting maps were thresholded at a cluster-wise threshold of *p* < 0.05, FWE-corrected, based on a height threshold of *p* < 0.005 (with the extent threshold of 47 voxels).

#### 2.4.2. Testing hypothesis 1b: Linking iFC change and trust in psychotherapists

To determine whether the supportive presence-related iFC change in the aDMN was related to participant’s trust in psychotherapists, we tested the correlation between the social presence effect and the trust scores, using SPSS Statistics (Version 26; IBM, Armonk, United States). We extracted and calculated the change of iFC in the social presence-sensitive DMN area (i.e., the significant cluster identified by the contrast social > non-social in the resting-state fMRI data) for each participant. We then tested the Kendall’s tau coefficient representing the relationship between the iFC difference values and trust in psychotherapist scores, across participants, using the significance level of *p* < 0.05. Kendall’s tau was used instead of Pearson’s r due to its robustness against potential outliers and non-normality (40).

#### 2.4.3. Testing hypothesis 2a: Linking iFC change and social reappraisal-related brain activation

To examine the relationship between the resting-state social presence effect and social reappraisal-related brain activation within the social reappraisal activation network (Hypothesis 2a), we tested a correlation between the social presence-induced iFC change in the dACC within the aDMN and the social reappraisal-related activation changes of the social reappraisal activation network. The social reappraisal network was derived from a paired *t*-test contrasting social reappraisal > social no-reappraisal conditions, centered on the time window of negative IAPS picture presentation (3). We used a whole-brain correction, FWE-corrected (*p* < 0.05) at the cluster level, based on a height threshold of *p* < 0.005 (with the extent threshold of 174 voxels).

SPSS Statistics was then used for the correlation analysis. We examined the correlation between the social presence-induced iFC difference values and the social reappraisal-related activation changes in each of the clusters, across participants, using Kendall’s coefficient tau and the significance level of *p* < 0.05. Kendall’s tau was used instead of the conventional Pearson’s correlation, because it is robust against potential outliers and non-normal data (40).

#### 2.4.4. Testing hypothesis 2b: Moderation analysis

To evaluate the behavioral relevance of the resting-state social presence effect for social reappraisal (Hypothesis 2b), we tested a moderated association between the social presence-related resting-state effect in the aDMN (X) and social reappraisal success (Y), with participants’ trust in psychotherapists used as the moderator (W). Social reappraisal success was calculated as the difference in emotional ratings (gathered at the end of every trial) between the social reappraisal and the social no-reappraisal conditions. We performed a moderation analysis in the form of multiple regression in SPSS Statistics, using the PROCESS macro (41). We specifically examined the moderation model as a whole, the amount of added variance by the interaction term (X*W), as well as zones of significance for the relationship between X (iFC change) and Y (social reappraisal success) depending on the moderator (trust in psychotherapists), using the Johnson-Neyman technique (41, 42).

### 2.5. Control experiment on an independent sample

To complement the main experiment and specifically further test the main manipulation of supportive social presence, a control resting-state experiment was additionally carried out on an independent sample of 23 healthy female participants. The aim of this experiment was to test whether the mere presence of another female would be enough to cause a change in iFC of the aDMN during rest. In this experiment, participants were either alone or accompanied by an unfamiliar female experimenter (one out of 4, randomly assigned) who sat beside them in the scanner room during the resting-state scan. The female experimenter had no supportive role and no normative title (such as “psychotherapist”). Participants were told that she had to be in the room throughout the scanning procedure due to a problem with the communication system. The same analysis routine was applied to assess the contrast of “simple” social presence vs. alone on the iFC of the aDMN. In short, from a group ICA, we identified the aDMN which exhibited highest spatial correlation (*r* = 0.42) with the established template of the aDMN (37) and a decent ICCASO score (>0.95). We additionally checked that the network indeed contained both MPFC and ACC, as expected. A paired *t*-test was employed to compare the iFC between the social and nonsocial conditions within the aDMN network, in both directions. The resulting maps were thresholded at a cluster-wise threshold of *p* < 0.05, FWE-corrected (based on a height threshold of *p* < 0.005).

## 3. Results

### 3.1. Psychotherapist’s presence enhanced iFC of the dACC within the aDMN

**Figure 2:**
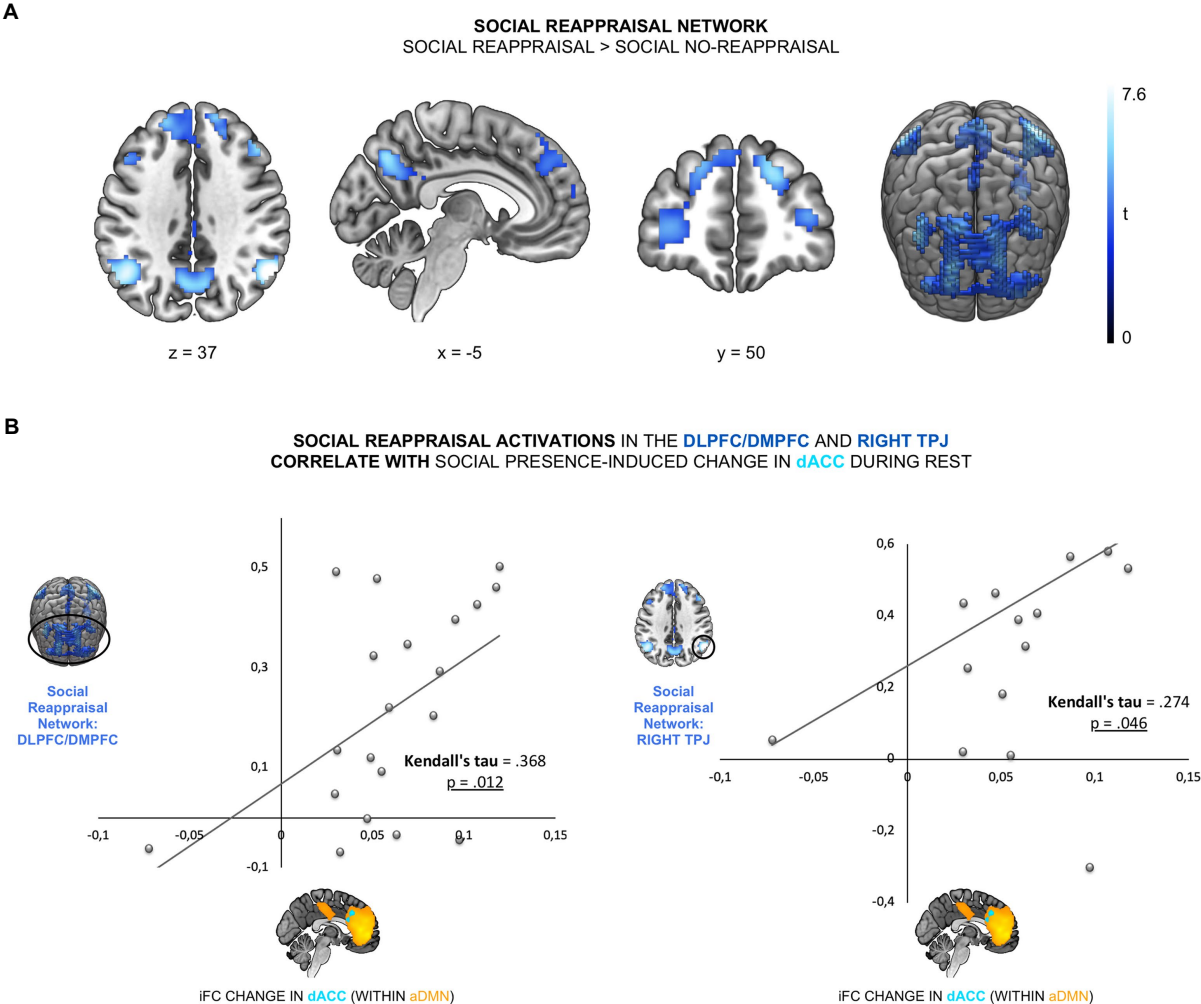
Psychotherapist’s presence enhanced iFC of dACC within the aDMN and was linked to trust in psychotherapists. **(A)** The contrast social > non-social exhibited an increased iFC of dACC (cyan; peak at [−3, 23, 37]) within the aDMN (orange). **(B)** This increase was positively correlated with participants’ general trust in psychotherapists (Kendall’s tau = 0.442, *p* = 0.005).

We first investigated the effect of perceived psychotherapist’s presence on the ongoing brain activity at rest, specifically iFC of the aDMN. ADMN was defined by a group ICA of resting-state fMRI data of 22 healthy female participants, acquired in two conditions: social (i.e., participants believed they were accompanied by a female psychotherapist sitting in a scanner-adjacent room and were connected with her *via* a camera-microphone system) and non-social (i.e., participants believed they were alone). IFC changes due to social presence were assessed by the contrast social versus non-social (in both directions), using paired *t*-tests. The contrast social > non-social exhibited an increased iFC of dACC (peak at [−3, 23, 37], cluster size *k* = 48, *p* < 0.05, FWE-corrected) within the aDMN (see Figure 2A; Supplementary Table 1).

To control for a possible anxiety-related confound—the possibility that changes in anxiety levels across the two experimental conditions might have (partly) caused the iFC change (53, 54)—participants’ anxiety levels were tested on a scale from 0 to 100 (0 = no anxiety; 100 = maximal anxiety) immediately prior to each of the two resting-state fMRI scans. Firstly, there was no significant difference in anxiety scores (paired *t*-test, *t*_21_ = −0.72, *p* = 0.48) between the social (*M* = 7.68, SD = 15.08) and the non-social condition (*M* = 9.77, SD = 14.35), suggesting that participants had comparable subjective levels of anxiety in both conditions. Secondly, we found no correlation between the degree of iFC change and differences in participants’ subjective anxiety levels across conditions (*r* = 0.09, *p* = 0.686). To further verify that the social presence effect was specific to the aDMN, the other three DMN sub-networks (i.e., anterior lateral DMN, posterior DMN and posterior dorsomedial DMN) were also tested. We observed no significant iFC change between the two conditions in any of the other three DMN sub-networks. Furthermore, no significant result was found for the contrast non-social > social in the aDMN, even at a liberal threshold (*p* < 0.001, uncorrected). Overall, results demonstrate that social presence was specifically associated with increased iFC of the dACC within the anterior medial DMN.

### 3.2. The social presence-related iFC effect was associated with participants’ trust in psychotherapists

To examine whether the social presence effect in the dACC was related to participants’ prior belief in psychotherapists’ trustworthiness, we correlated iFC changes in the dACC with participants’ general trust in psychotherapists. We found that social presence-related increase of dACC iFC within the aDMN was positively correlated with participants’ trust in psychotherapists (Kendall’s *tau* = 0.442, *p* = 0.005; Figure 2B). This demonstrates that those participants who, in general, trusted psychotherapists more (i.e., believed to a higher extent that psychotherapists are reliable and supportive), displayed a stronger neural sensitivity to social presence in the aDMN during rest.

### 3.3. The social presence-related iFC change in the dACC was linked with the social reappraisal-related activation increases in the DMPFC/DLPFC and right TPJ

**Figure 3:**
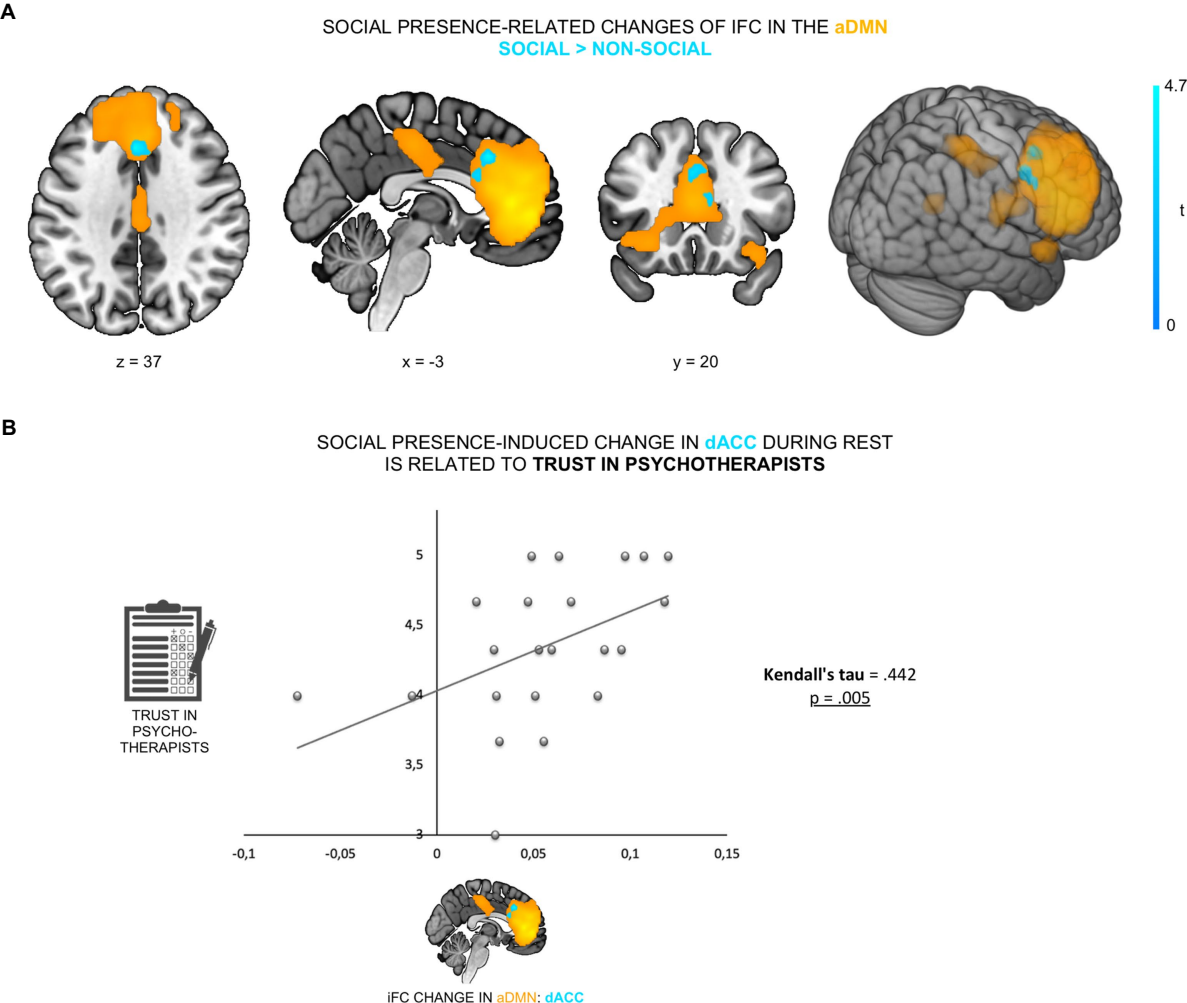
The social presence-related effect in the aDMN was linked to social reappraisal-related brain activations. **(A)** The social reappraisal network was derived from the contrast social reappraisal > social no-reappraisal during negative picture viewing of the social reappraisal task, whole-brain FWE-corrected (*p* < 0.05) at the cluster levels, based on a height threshold of 0.005. **(B)** The social presence-related change in the dACC was correlated with the social reappraisal-related activation changes in the DLPFC/DMPFC (Kendall’s tau = 0.368, *p* = 0.012) and right TPJ (Kendall’s tau = 0.274, *p* = 0.046).

To test the link between the effect of social presence on aDMN and social reappraisal during negative emotions, we examined the relationship between the social presence effect and both social reappraisal-related brain activation and social reappraisal effectiveness. The first association gives us hints about the neural mechanism behind the effect of social presence on social reappraisal and the second association tests the functional outcome of social presence DMN effect. To first test the relationship between the social presence effect in the aDMN and the social reappraisal activation network (Hypothesis 2a), we identified the social reappraisal network. The contrast social reappraisal > social no-reappraisal revealed 5 significant clusters: the frontal cluster, covering bilateral dorsomedial prefrontal cortex (DMPFC) and dorsolateral prefrontal cortex (DLPFC) regions, left and right TPJ clusters, the precuneus cluster, and a left cerebellum cluster (see Figure 3A; Supplementary Table 2). We then examined the relationship between the social presence-sensitive dACC within the aDMN and the five cluster of the social reappraisal activation network. We found a significant positive correlation between the iFC difference values and the social reappraisal-related activation changes in the bilateral DMPFC/DLPFC cluster (Kendall’s *tau* = 0.368, *p* = 0.012) and the right TPJ cluster, across participants (Kendall’s *tau* = 0.274, *p* = 0.046; see Figure 3B). Results thus show a positive relationship between the social presence and the social reappraisal effects at the neural level, such that the stronger the change in the dACC connectivity within aDMN during rest while in the presence of the psychotherapist, the stronger the activation increase in the social reappraisal activation network – specifically in the bilateral DMPFC, DLPFC, and right TPJ.

### 3.4. The social presence-induced iFC change was linked to the success of social reappraisal, moderated by trust in psychotherapists

**Figure 4:**
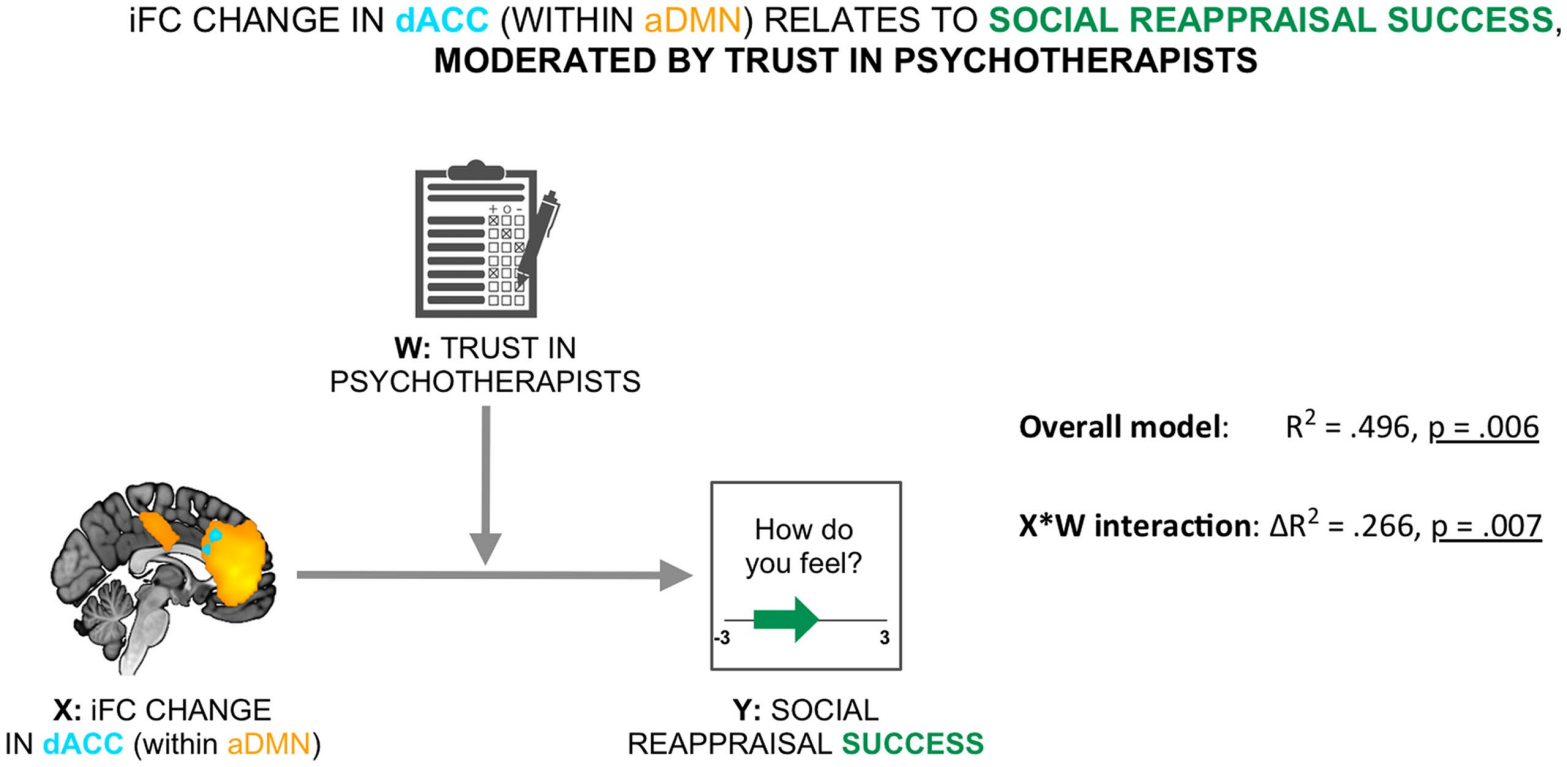
Social reappraisal effectiveness was related to the social presence-related change in the aDMN, moderated by trust in psychotherapists. The iFC change in dACC within the aDMN was related to social reappraisal success (calculated as the difference in emotional ratings between the social reappraisal and the social no-reappraisal conditions), moderated by trust in psychotherapists (overall model: *R*^2^ = 496, *p* = 0.006; X*W interaction: Δ*R*^2^ = 0.266, *p* = 0.007).

To evaluate the relevance of the resting-state social presence effect for social reappraisal success, we tested a moderated association between the social presence-related resting-state effect in the dACC within the aDMN (X) and social reappraisal success (Y), with participants’ trust in psychotherapists used as the mediator (W) (see Figure 4). We found that the moderation model as a whole explained a significant amount of variance in social reappraisal success (*R^2^* = 0.496, *F* (3,18) = 5.896, *p* = 0.006). We further observed that the interaction term (X*W), representing the moderated effect, accounted for a significant proportion of the variance in social reappraisal success (Δ*R^2^* = 0.266, *F* (1,18) = 9.474, *p* = 0.007; see Figure 4). Results demonstrated that the relationship between the iFC change in dACC during rest and social reappraisal success was indeed moderated by participants’ general trust in psychotherapists.

The Johnson–Neyman technique identified a single zone of significance at the lower end of the trust scores: there was a positive relationship between the social presence-induced iFC change in the dACC and social reappraisal success, which remained significant until the trust score of 4.18 (36th percentile; *b* = 4.307, *t* (36) = 2.101, *p* = 0.05), but was no longer significant for the highest trust levels (between 4.18 and 5).

### 3.5. Control experiment results: No effect for social presence of stranger

To further test the main manipulation of supportive social presence, an additional resting-state experiment was conducted on an independent sample of 23 participants, to test whether the findings of the main experiment were indeed due to the supportive presence of a psychotherapist and not the mere presence of a person. When participants were accompanied by an unfamiliar female experimenter, a paired *t*-test on the iFC of the aDMN showed no significant activations for the contrast social > non-social, as well as the reverse contrast of non-social > social.

## 4. Discussion

The current study investigated the impact of social presence-related changes within the brain’s DMN on neural and behavioral correlates of social reappraisal. We firstly examined whether the presence of a psychotherapist will induce changes in the aDMN functional connectivity *during rest* and secondly asked whether these changes might influence social reappraisal-related brain activations and social reappraisal success *during negative emotions*. In the first part of the experiment, resting-state functional connectivity of infra-slow BOLD fluctuations was examined in (a) the social condition, in which participants believed they were accompanied by a psychotherapist, and (b) the non-social condition, which they underwent alone. The second part of the experiment, in contrast, involved two task conditions: social reappraisal, during which the psychotherapist actively helped participants to regulate their evoked negative emotions, and social no-reappraisal, during which she told participants to attend to the emotional stimuli and respond naturally. The findings reveal, for the first time, that social presence—a salient social cue—can produce significant changes in the resting human brain. Results further demonstrate that our brain’s sensitivity to another person during rest can influence, depending on trust, both neural activations and the effectiveness of social emotion regulation.

### 4.1. Psychotherapist’s presence and the aDMN

#### 4.1.1. Psychotherapist’s presence enhanced iFC of the dACC within the aDMN

Confirming hypothesis 1a, social presence increased iFC, the correlations between spontaneous fluctuations of the BOLD signal at rest (Figure 3A). Even though iFC is generally seen as a stable marker, mapping the brain’s functional organization (10, 11, 43), studies have shown that iFC shows changes after cognitive training (12, 44, 45), as well as across time and mental states within an individual (10, 13, 46). DMN connectivity, specifically, can be modulated by external input, such as spoken narratives (6, 14). In agreement with these studies yet distinct from previous findings, our data demonstrated that the brain’s iFC was changed by the supportive social presence of a trustworthy other. Our results showed that a brief priming of social presence led to a significant change in ongoing brain activity, consistent with a recent observation that hand holding by a supportive other modulated the brain activity during rest intervals of an emotional stimulation task (24). Interestingly, our data indicate that primed social presence could induce changes in ongoing brain activity even in the absence of physical connection, similar to what had previously been observed in studies on spousal support by means of photographs (47, 48). We speculate that the social presence scenario might have primed socially oriented thoughts in the form of a “social cognitive map” (18, 49) in which the self and presented figure become connected. Unconstraint thoughts would shape the spontaneous introspective processes during a “free thinking” state, which ultimately determined a unique brain state at rest, relative to being alone. This is compatible with the Social Baseline Theory, which suggests that the human brain is by default adapted by the availability of social resources and that proximity to supportive others should thus represent a fundamentally different brain state, since being alone represents comparatively more challenges for the individual’s brain (50, 51). This does not, however, indicate that the difference between conditions was driven by increases in anxiety during the alone condition, as the iFC change was not related to participants’ anxiety levels, ruling out stress-related explanations of the brain activity’s modulation (52, 53).

Considering the main manipulation of our study—supportive presence versus alone, an alternative interpretation of our finding could be that the mere presence of a person (any person) can induce changes in the brain’s iFC. To address this, a control experiment was carried out, on an independent sample, in which participants were either alone or accompanied by an unfamiliar female experimenter, without an explicit supportive role and without a supportive/trustworthy normative title (e.g., “psychotherapist”). The presence of the stranger was as neutral as possible—they were also female, and accompanying the participant in the scanner room due to a “faulty communication system.” Results confirmed that the mere presence of a person, an unthreatening stranger, was not sufficient to induce changes in iFC of the aDMN during rest. This supports the main interpretation of the current study, namely that a supportive presence of a trustworthy other can cause changes in intrinsic brain connectivity.

Regarding the effect’s spatial extent, we confirmed that social presence increased iFC within the aDMN (see Figure 3A). On the whole, the DMN is seen as an integrator of external inputs and intrinsic information that, as such, supports our social lives and social interactions (6). The DMN is also interpreted as a neural system for random episodic thoughts about the self and others (4, 5, 55), with aDMN thought to be preferentially involved in socially oriented thoughts (56–58). A large body of literature indeed reports that the key regions of the aDMN, including the MPFC, OFC, and ACC, are frequently engaged in a range of social cognitive processes, from mentalization and perspective taking to empathy (19, 59, 60). Furthermore, increased activation of the MPFC and ventromedial PFC (VMPFC) has been observed when participants viewed photos of their partner during a stressful situation (48, 61), while we previously observed a reduction of activity in anterior DMN areas (VMPFC, OFC and ACC) during the perceived supportive presence of a psychotherapist during negative emotions (62). Consistently, our data showed that the aDMN was indeed susceptible to the sense of being with a supportive other during rest, primed through a brief social scenario.

In the present study, the change of iFC within the aDMN was centered on the dACC, which showed increased iFC with other regions of the aDMN during social presence. As well as to the affective component of physical pain (63, 64), dACC responds to social exclusion and lack/absence of social support (22, 23, 62, 65). Even without direct negative stimulation (during resting periods), voxels in the dACC were previously shown to respond with higher response magnitudes while alone compared to holding a friend’s hand (24), coinciding with our result. Among other proposed functions, dACC is seen as the key region for mental representations of another’s yet unknown actions, such as anticipating another’s intentions or state of mind and incorporating them into one’s actions during a mutual interaction (19, 66). A recent animal study found that the neurons in the monkey’s dACC selectively encoded another monkey’s unknown decision in a prisoner’s dilemma game, and disruption of the dACC biased mutually beneficial interactions between the monkeys (66). Accordingly, in the present study, the sensitivity of the dACC to social presence may reflect a similar mental process in which participants were aware of the psychotherapist’s presence in a shared context even during the resting state. In accordance, previous human studies have provided evidence for the essential role of the dACC in both encoding social interest and enacting interactive behavior in social interactions. For example, the activation of the ACC (including the dorsal part) tracked the weight of interest assigned to another social agent but not that assigned to comparable nonsocial variables (67). Similarly, the medial bank of middle cingulate regions (covering the dACC) was involved in social exchange, and this activation only occurred in the presence of a responsive social partner (68). Finally, iFC between ACC (including dACC) and other typical social brain regions (e.g., DMPFC and superior temporal sulcus) increased with social group size in humans (57, 69). Our data confirm this and further suggest that the sense of being accompanied by the psychotherapist induced by a simple scenario is a powerful trigger to foster a social connection to another and motivate us to interact with them.

#### 4.1.2. The social presence-related iFC effect was associated with participants’ trust in psychotherapists

In line with hypothesis 1b, data showed that the iFC change was associated with participants’ trust in psychotherapists, such that participants who trusted psychotherapists more displayed a stronger neural sensitivity to social presence (Figure 3B). Interpersonal trust in psychotherapists reflects the extent to which participants believe that psychotherapists are reliable and supportive when needed in general (36). Interpersonal trust is critical for determining how people respond to interpersonal events and behave in social contexts. For instance, individuals with high (vs. low) interpersonal trust are more likely to be engaged in social interaction and sustain benevolent intentions when they encounter uncertainty (70). Furthermore, previous behavioral studies found generalized interpersonal trust to be associated with help-seeking behaviors and the amounts of received support (71, 72). In line with this, our finding suggests that participants’ appraisal of the presented figure plays a key role in the social presence effect, indicating that the level of general trust in psychotherapists relates to the level of positive influence a specific psychotherapist will have on an individual. Importantly, our data suggest that trust might play an important role in social regulation by previously unknown individuals with professional or trustworthy labels (e.g., “psychotherapist”), which would be worth replicating and investigating further. In a general sense, however, the result is in line with studies showing that the relationship with the regulator (both the type of relationship and the quality of the relationship) largely affects the success of social emotion or pain regulation (15, 16).

### 4.2. Psychotherapist’s presence, aDMN, and social reappraisal

#### 4.2.1. The social presence-induced iFC change was linked with the social reappraisal-related brain activations

In the current study, social reappraisal-related activation increases within the social reappraisal activation network, specifically bilateral DMPFC/DLPFC and right TPJ, were positively related to the social presence-related iFC change in the dACC, confirming hypothesis 2a (Figure 2B). Focusing on the neural level, this relationship augments the link between social presence and social reappraisal effects, such that the stronger the change in dACC due to the psychotherapist’s presence, the stronger the social reappraisal-related activations in the DMPFC/DLPFC and right TPJ. This is in line with a recent study where the reappraisal of negative images was stronger when initiated by a close friend compared to a stranger or participants themselves (31). Results highlight, to our knowledge for the first time, a positive link between the two social regulation processes, social presence and social reappraisal, as changes in the dACC due to supportive psychotherapist’s presence and changes in the frontal cortex and the right TPJ due to social reappraisal by the same psychotherapist were positively correlated.

#### 4.2.2. The social presence-induced iFC change was linked to the success of social reappraisal, moderated by trust in psychotherapists

As was anticipated by hypothesis 2b, the social presence-induced iFC change in the dACC within the aDMN was positively related to social reappraisal success, with trust in psychotherapists acting as a moderator (Figure 4). After examining the interaction with the Johnson-Neyman technique, the positive impact of the psychotherapist during resting state on social reappraisal success remained significant for trust scores up to 4.18. We propose that those participants who trusted psychotherapists less in general approached the study, the specific psychotherapist, and the social regulation with more uncertainty and were thus more influenced by the unfolding of events, especially the personal meeting with the therapist and the resting state scan. In the event that they were less susceptible to her presence during the resting state scan, they (on average) also showed less susceptibility to her social reappraisal of participant’s negative emotions. In the case that they were influenced by her presence during the resting state scan, the psychotherapist was also more able to regulate their negative emotions in the second part of the experiment. In contrast, participants that trusted psychotherapists to a very high extent (i.e., had a score of 4.2 or higher out of 5) were seemingly not constrained by the impression of this specific therapist in relation to her effectiveness in regulating their emotions. Importantly, this is not to be interpreted as a lack of relationship between trust in psychotherapists and social reappraisal success. In fact, the more participants trusted psychotherapists in general, the higher the social reappraisal effectiveness (Kendall’s *tau* = 0.346, *p* = 0.017). The result might in contrast point to a crucial distinction between general trust in psychotherapists and trust in a specific psychotherapist. General trust in psychotherapists separately enhanced both the resting state effect of the psychotherapist’s presence and the effect of social reappraisal. However, with regard to the influence of resting state effect on the social reappraisal effectiveness of the same psychotherapist, the crucial parameter to enhance this connection might be trust in the specific psychotherapist. To test this hypothesis, future similar studies should measure both types of trust.

### 4.3. Implications for psychotherapy and affective disorders

Results of the study have important implications for everyday interpersonal relationships, pointing to a possible interdependence of two social processes, social presence and social reappraisal. However, the findings are especially relevant for the therapist-client relationship within the context of psychotherapy, which focuses on interaction-focused interventions and often involves some form of social reappraisal (73–75). Our findings suggest that there are at least two key factors that might influence the success of deliberate cognitive social emotion regulation by the psychotherapist, likely also important for the success of the therapy in general. The first factor is general trust in psychotherapists, which points to the significance of increasing trust in psychotherapists in the general population. The second factor is supportive social presence. Especially for those clients or patients that do not generally trust psychotherapists, it is crucial to consider their susceptibility or awareness of being accompanied and cared for by the specific psychotherapist, as it might influence the therapeutic success. Future studies could investigate how long-term the effect is and which intervention (e.g., raising trust within the relationship, intensifying the initial therapeutic stage to build the connection) might be most successful.

It is both interesting and relevant to also consider the current findings in relation to affective disorders, including depression and anxiety. On the one hand, emotion dysregulation plays a central role in these disorders (74, 76, 77), while on the other hand, affective disorders have been characterized by altered neural functioning of the default mode network (78, 79). As such, improving negative emotion regulation and reactivity is crucial for the success of psychotherapy (80), yet the involvement of the default mode network in both supportive social presence and social reappraisal might make it more difficult or even unlikely for patients with affective disorders to benefit from the two social regulation strategies. Further studies could examine the link between default mode network impairment and receptiveness of social regulation, as well as the possible lack of the relationship between social presence and social reappraisal in individuals with affective disorders.

### 4.4. Limitations

The current study’s results should be interpreted with some reservations. Firstly, as the first examination of the relationship between social presence and social reappraisal, the study included a moderate sample and followed a non-preregistered hypothesis-driven approach. We additionally only recruited females, as a compromise between the signal-to-noise ratio (related to sample homogeneity) and generalizability of our findings. The decision was made in light of reported gender differences in the neural substrates of emotional processing (32, 33) and emotion regulation (34, 35). Based on existing research only focusing on intrapersonal emotion regulation, it is not clear whether male participants would show a different effect in the aDMN in response to supportive psychotherapist’s presence or her regulation of negative emotional responses, both in terms of strength and location. Another interesting question relates to the gender of the regulator. Further studies are needed, characterizing gender differences in social support and social reappraisal, both in relation to the regulator and the target person. It is also worth noting that recruitment within the medical faculty resulted in an overrepresentation of medicine students in the sample, which likely resulted in the high average trust in psychotherapists score reported in the study. In terms of sample size, it is crucial to note that smaller sample sizes are related to less statistical power, which generally represents an important problem within the neuroimaging community, as strict multiple comparison correction standards also greatly reduce power in many imaging experiments (81, 82). Nevertheless, power and interpretability can also be increased by robust specific hypotheses combined with a within-subjects design, many trial repetitions, and a ROI-based approach, which have all been considered and followed in the current study (82). Further studies that would replicate the findings in a larger, more diverse, sample are greatly encouraged. Similarly, future investigations could use a real-life social interaction, as well as more naturalistic emotional stimuli, increasing the ability to generalize results to everyday social interactions. Even though the current study is one of the few enlisting the help of a psychotherapist as the regulator, it would be very interesting to investigate the found effects in a wider setting and test in what way the psychotherapist’s presence and reappraisal differ from the social regulation by a partner or a complete stranger (without a normative title). Finally, due to the resting state design, we did not obtain emotional ratings during social presence, and only obtained momentary ratings of general emotional valence during social reappraisal. It would be highly interesting to more thoroughly investigate the underlying processes of social presence and social reappraisal, especially pertaining to the brain-behavior link.

## 5. Conclusion

The present study demonstrated, for the first time, that social presence can alter ongoing brain activity during rest and that this change is linked to both social reappraisal-related brain activations and social reappraisal success. Firstly, our findings highlight that the brain structure of dACC and its functionally related areas in the aDMN are associated with the baseline social presence effect. Secondly, our findings highlight a positive association between two social processes: the supportive social presence, centered on the aDMN, and the social reappraisal of negative emotions, specifically (a) social reappraisal-related activations in the bilateral DMPFC/DLPFC and right TPJ and (b) behavioral effectiveness of social reappraisal, moderated by trust in psychotherapists. The findings have important implications for understanding the functional roles of aDMN and TPJ in social cognition, and for investigating the impact of social functioning in relevant psychopathological conditions. Last but not least, they also call for a careful control of context in resting state imaging studies.

## Data availability statement

The raw data supporting the conclusions of this article will be made available by the authors, without undue reservation.

## Ethics statement

The studies involving human participants were reviewed and approved by Ethics Committee of the Klinikum rechts der Isar at the Technical University of Munich. The patients/participants provided their written informed consent to participate in this study. Written informed consent was obtained from the individual(s) for the publication of any potentially identifiable images or data included in this article.

## Author contributions

XX, CS, and SMB contributed to conception and design of the study. XX, TB, and SMB performed the experiment/data collection. XX, TB, SZ, MH, CS, and SMB performed the statistical analyses. XX and SMB wrote the first draft of the manuscript. SZ, MH, and CS wrote sections of the manuscript. All authors contributed to manuscript revision and read and approved the submitted version.

## Funding

This work was supported by the Slovenian Research Agency (ARRS J3-2525) to SMB, SZ, and MH.

## Conflict of interest

The authors declare that the research was conducted in the absence of any commercial or financial relationships that could be construed as a potential conflict of interest.

## Publisher’s note

All claims expressed in this article are solely those of the authors and do not necessarily represent those of their affiliated organizations, or those of the publisher, the editors and the reviewers. Any product that may be evaluated in this article, or claim that may be made by its manufacturer, is not guaranteed or endorsed by the publisher.

## Footnotes

^1^ https://trendscenter.org/software/#gica/

^2^ http://research.ics.aalto.fi/ica/icasso/

^3^ https://trendscenter.org/data/

## References

[1] Grecucci A Theuninck A Frederickson J Job R. “Mechanisms of social emotion regulation: from neuroscience to psychotherapy,” in Handbook of Emotion Regulation: Processes, Cognitive Effects and Social Consequences. ed. Bryant M. L. (Nova Publisher), (2015).

[2] Reeck C Ames DR Ochsner KN. The social regulation of emotion: an integrative, cross-disciplinary model. Trends Cogn Sci. (2015) 20:47–63. doi: 10.1016/j.tics.2015.09.003, PMID: 26564248 PMC5937233

[3] Xie X Mulej Bratec S Schmid G Meng C Doll A Wohlschläger A. How do you make me feel better? Social cognitive emotion regulation and the default mode network. NeuroImage. (2016) 134:270–80. doi: 10.1016/j.neuroimage.2016.04.015, PMID: 27095057

[4] Raichle ME. The brain’s default mode network. Annu Rev Neurosci. (2015) 38:433–47. doi: 10.1146/annurev-neuro-071013-014030 25938726

[5] Spreng RN Andrews-Hanna JR Toga AW. “The default network and social cognition,” Waltham: Academic Press (2015). 165–169. Available at: http://www.sciencedirect.com/science/article/pii/B9780123970251001731

[6] Yeshurun Y Nguyen M Hasson U. The default mode network: where the idiosyncratic self meets the shared social world. Nat Rev Neurosci. (2021) 22:181–92. doi: 10.1038/s41583-020-00420-w, PMID: 33483717 PMC7959111

[7] Fox MD Snyder AZ Vincent JL Corbetta M Essen DCV Raichle ME. The human brain is intrinsically organized into dynamic, anticorrelated functional networks. Proc Natl Acad Sci U S A. (2005) 102:9673–8. doi: 10.1073/pnas.0504136102, PMID: 15976020 PMC1157105

[8] Biswal BB Mennes M Zuo X-N Gohel S Kelly C Smith SM. Toward discovery science of human brain function. Proc Natl Acad Sci U S A. (2010) 107:4734–9. doi: 10.1073/pnas.0911855107, PMID: 20176931 PMC2842060

[9] Cole MW Bassett DS Power JD Braver TS Petersen SE. Intrinsic and task-evoked network architectures of the human brain. Neuron. (2014) 83:238–51. doi: 10.1016/j.neuron.2014.05.014, PMID: 24991964 PMC4082806

[10] Laumann TO Gordon EM Adeyemo B Snyder AZ Joo SJ Chen M-Y. Functional system and areal organization of a highly sampled individual human brain. Neuron. (2015) 87:657–70. doi: 10.1016/j.neuron.2015.06.037, PMID: 26212711 PMC4642864

[11] Gratton C Laumann TO Nielsen AN Greene DJ Gordon EM Gilmore AW. Functional brain networks are dominated by stable group and individual factors, not cognitive or daily variation. Neuron. (2018) 98:e5:439–52. doi: 10.1016/j.neuron.2018.03.035, PMID: 29673485 PMC5912345

[12] Bueichekú E Miró-Padilla A Ávila C. Resting-state fMRI detects the effects of learning in short term: a visual search training study. Hum Brain Mapp. (2019) 40:2787–99. doi: 10.1002/hbm.24560, PMID: 30859709 PMC6865379

[13] Geerligs L Rubinov M Cam-Can HRN. State and trait components of functional connectivity: individual differences vary with mental state. J Neurosci Official J Soc Neurosci. (2015) 35:13949–61. doi: 10.1523/jneurosci.1324-15.2015 PMC4604231 26468196

[14] Simony E Honey CJ Chen J Lositsky O Yeshurun Y Wiesel A. Dynamic reconfiguration of the default mode network during narrative comprehension. Nat Commun. (2016) 7:12141. doi: 10.1038/ncomms12141, PMID: 27424918 PMC4960303

[15] Krahé C Springer A Weinman JA Fotopoulou A. The social modulation of pain: others as predictive signals of salience – a systematic review. Front Hum Neurosci. (2013) 7:386. doi: 10.3389/fnhum.2013.00386, PMID: 23888136 PMC3719078

[16] Eisenberger NI. An empirical review of the neural underpinnings of receiving and giving social support: implications for health. Psychosom Med. (2013) 75:545–56. doi: 10.1097/psy.0b013e31829de2e7, PMID: 23804014 PMC3763941

[17] Hostinar CE Gunnar MR. Social support can buffer against stress and shape brain activity. AJOB Neurosci. (2015) 6:34–42. doi: 10.1080/21507740.2015.1047054, PMID: 26478822 PMC4607089

[18] Peer M Salomon R Goldberg I Blanke O Arzy S. Brain system for mental orientation in space, time, and person. Proc National Acad Sci U S A. (2015) 112:11072–7. doi: 10.1073/pnas.1504242112, PMID: 26283353 PMC4568229

[19] Rushworth MFS Mars RB Sallet J. Are there specialized circuits for social cognition and are they unique to humans? Curr Opin Neurobiol. (2013) 23:436–42. doi: 10.1016/j.conb.2012.11.013, PMID: 23290767

[20] Delgado MR Beer JS Fellows LK Huettel SA Platt ML Quirk GJ. Viewpoints: dialogues on the functional role of the ventromedial prefrontal cortex. Nat Neurosci. (2016) 19:1545–52. doi: 10.1038/nn.4438, PMID: 27898086

[21] Rushworth MFS Behrens TEJ Rudebeck PH Walton ME. Contrasting roles for cingulate and orbitofrontal cortex in decisions and social behaviour. Trends Cogn Sci. (2007) 11:168–76. doi: 10.1016/j.tics.2007.01.004, PMID: 17337237

[22] Eisenberger NI Taylor SE Gable SL Hilmert CJ Lieberman MD. Neural pathways link social support to attenuated neuroendocrine stress responses. NeuroImage. (2007) 35:1601–12. doi: 10.1016/j.neuroimage.2007.01.038, PMID: 17395493 PMC2710966

[23] Eisenberger NI. Broken hearts and broken bones: a neural perspective on the similarities between social and physical pain. Curr Dir Psychol Sci. (2012) 21:42–7. doi: 10.1177/0963721411429455

[24] Zhang T Li F Beckes L Coan JA. A semi-parametric model of the hemodynamic response for multi-subject fMRI data. NeuroImage. (2013) 75:136–45. doi: 10.1016/j.neuroimage.2013.02.048, PMID: 23473935

[25] Jennings L Skovholt TM. The cognitive, emotional, and relational characteristics of master therapists. J Couns Psychol. (1999) 46:3–11. doi: 10.1037//0022-0167.46.1.3

[26] Pletzer JL Sanchez X Scheibe S. Practicing psychotherapists are more skilled at downregulating negative emotions than other professionals. Psychotherapy Chic Ill. (2015) 52:346–50. doi: 10.1037/a0039078, PMID: 25938790

[27] Crits-Christoph P Rieger A Gaines A Gibbons MBC. Trust and respect in the patient-clinician relationship: preliminary development of a new scale. Bmc Psychology. (2019) 7:91. doi: 10.1186/s40359-019-0347-3, PMID: 31888759 PMC6937966

[28] Peschken W Johnson M. Therapist and client trust in the therapeutic relationship. Psychother Res. (1997) 7:439–47. doi: 10.1080/10503309712331332133

[29] Yeshurun Y Swanson S Simony E Chen J Lazaridi C Honey CJ. Same story, different story. Psychol Sci. (2016) 28:307–19. doi: 10.1177/0956797616682029, PMID: 28099068 PMC5348256

[30] Lee H Bellana B Chen J. What can narratives tell us about the neural bases of human memory? Curr Opin Behav Sci. (2020) 32:111–9. doi: 10.1016/j.cobeha.2020.02.007

[31] Morawetz C Berboth S Bode S. With a little help from my friends: the effect of social proximity on emotion regulation-related brain activity. NeuroImage. (2021) 230:117817. doi: 10.1016/j.neuroimage.2021.117817, PMID: 33529742

[32] Whittle S Yücel M Yap MBH Allen NB. Sex differences in the neural correlates of emotion: evidence from neuroimaging. Biol Psychol. (2011) 87:319–33. doi: 10.1016/j.biopsycho.2011.05.003 21600956

[33] Moriguchi Y Touroutoglou A Dickerson BC Barrett LF. Sex differences in the neural correlates of affective experience. Soc Cogn Affect Neur. (2014) 9:591–600. doi: 10.1093/scan/nst030, PMID: 23596188 PMC4014103

[34] McRae K Ochsner KN Mauss IB Gabrieli JJD Gross JJ. Gender differences in emotion regulation: an fMRI study of cognitive reappraisal. Group Process Intergroup Relat. (2008) 11:143–62. doi: 10.1177/1368430207088035, PMID: 29743808 PMC5937254

[35] Domes G Schulze L Böttger M Grossmann A Hauenstein K Wirtz PH. The neural correlates of sex differences in emotional reactivity and emotion regulation. Hum Brain Mapp. (2010) 31:758–69. doi: 10.1002/hbm.20903, PMID: 19957268 PMC6871188

[36] Kassenbaum UB. Interpersonelles Vertrauen: Entwicklung eines Inventars zur Erfassung spezifischer Aspekte des Konstrukts. SUB Hamburg. (2004).

[37] Allen EA Erhardt EB Damaraju E Gruner W Segall JM Silva RF. A baseline for the multivariate comparison of resting-state networks. Front Syst Neurosci. (2011) 5:2. doi: 10.3389/fnsys.2011.00002, PMID: 21442040 PMC3051178

[38] Abou-Elseoud A Starck T Remes J Nikkinen J Tervonen O Kiviniemi V. The effect of model order selection in group PICA. Hum Brain Mapp. (2009) 31:1207–16. doi: 10.1002/hbm.20929, PMID: 20063361 PMC6871136

[39] Kiviniemi V Starck T Remes J Long X Nikkinen J Haapea M. Functional segmentation of the brain cortex using high model order group PICA. Hum Brain Mapp. (2009) 30:3865–86. doi: 10.1002/hbm.20813, PMID: 19507160 PMC6870574

[40] Xu W Hou Y Hung YS Zou Y. Comparison of Spearman’s rho and Kendall’s tau in normal and contaminated normal models. Arxiv. (2010)

[41] Hayes AF. Introduction to mediation, moderation, and conditional process analysis, second edition: a regression-based approach. New York: Guilford Press (2017). 692.

[42] Memon MA Cheah J-H Ramayah T Ting H Chuah F Cham TH. Moderation analysis: issues and guidelines. J Appl Struct Equ Model. (2019) 3:i–xi. doi: 10.47263/jasem.3(1)01

[43] Raichle ME. The restless brain: how intrinsic activity organizes brain function. Philos Trans R Society London B: Biol Sci. (2015) 370:20140172–2. doi: 10.1098/rstb.2014.0172, PMID: 25823869 PMC4387513

[44] Thompson TW Waskom ML Gabrieli JDE. Intensive working memory training produces functional changes in large-scale Frontoparietal networks. J Cognitive Neurosci. (2016) 28:575–88. doi: 10.1162/jocn_a_00916, PMID: 26741799 PMC5724764

[45] Tang Y-Y Posner MI. Training brain networks and states. Trends Cogn Sci. (2014) 18:345–50. doi: 10.1016/j.tics.2014.04.002 24816329

[46] Kucyi A Davis KD. The dynamic pain connectome. Trends Neurosci. (2015) 38:86–95. doi: 10.1016/j.tins.2014.11.006 25541287

[47] Master SL Eisenberger NI Taylor SE Naliboff BD Shirinyan D Lieberman MD. A picture’s worth: partner photographs reduce experimentally induced pain. Psychol Sci. (2009) 20:1316–8. doi: 10.1111/j.1467-9280.2009.02444.x 19788531

[48] Eisenberger NI Master SL Inagaki TK Taylor SE Shirinyan D Lieberman MD. Attachment figures activate a safety signal-related neural region and reduce pain experience. Proc Natl Acad Sci U S A. (2011) 108:11721–6. doi: 10.1073/pnas.1108239108, PMID: 21709271 PMC3136329

[49] Epstein RA Patai EZ Julian JB Spiers HJ. The cognitive map in humans: spatial navigation and beyond. Nat Neurosci. (2017) 20:1504–13. doi: 10.1038/nn.4656, PMID: 29073650 PMC6028313

[50] Coan JA Sbarra DA. Social baseline theory: the social regulation of risk and effort. Curr Opin Psychol. (2015) 1:87–91. doi: 10.1016/j.copsyc.2014.12.021, PMID: 25825706 PMC4375548

[51] Beckes L Sbarra DA. Social baseline theory: state of the science and new directions. Curr Opin Psychol. (2021) 43:36–41. doi: 10.1016/j.copsyc.2021.06.004, PMID: 34280688

[52] Sylvester CM Corbetta M Raichle ME Rodebaugh TL Schlaggar BL Sheline YI. Functional network dysfunction in anxiety and anxiety disorders. Trends Neurosci. (2012) 35:527–35. doi: 10.1016/j.tins.2012.04.012, PMID: 22658924 PMC3432139

[53] Seeley WW Menon V Schatzberg AF Keller J Glover GH Kenna H. Dissociable intrinsic connectivity networks for salience processing and executive control. J Neurosci. (2007) 27:2349–56. doi: 10.1523/jneurosci.5587-06.2007, PMID: 17329432 PMC2680293

[54] Kim MJ Gee DG Loucks RA Davis FC Whalen PJ. Anxiety dissociates dorsal and ventral medial prefrontal cortex functional connectivity with the amygdala at rest. Cerebral Cortex. (2011) 21:1667–1673. doi: 10.1093/cercor/bhq237 21127016 PMC3116741

[55] Andrews-Hanna JR Reidler JS Sepulcre J Poulin R Buckner RL. Functional-anatomic fractionation of the Brain’s default network. Neuron. (2010) 65:550–62. doi: 10.1016/j.neuron.2010.02.005, PMID: 20188659 PMC2848443

[56] Amft M Bzdok D Laird AR Fox PT Schilbach L Eickhoff SB. Definition and characterization of an extended social-affective default network. Brain Struct Funct. (2015) 220:1031–49. doi: 10.1007/s00429-013-0698-0, PMID: 24399179 PMC4087104

[57] Mars RB Neubert F-X Noonan MP Sallet J Toni I Rushworth MFS. On the relationship between the “default mode network” and the “social brain”. Front Hum Neurosci. (2012) 6:189. doi: 10.3389/fnhum.2012.00189, PMID: 22737119 PMC3380415

[58] Schilbach L Bzdok D Timmermans B Fox PT Laird AR Vogeley K. Introspective minds: using ALE meta-analyses to study commonalities in the neural correlates of emotional processing, Social & Unconstrained Cognition. PLoS One. (2012) 7:e30920. doi: 10.1371/journal.pone.0030920, PMID: 22319593 PMC3272038

[59] Adolphs R. The social brain: neural basis of social knowledge. Annu Rev Psychol. (2009) 60:693–716. doi: 10.1146/annurev.psych.60.110707.163514, PMID: 18771388 PMC2588649

[60] Frith CD Frith U. Social cognition in humans. Curr Biol. (2007) 17:R724–32. doi: 10.1016/j.cub.2007.05.068 17714666

[61] Younger J Aron A Parke S Chatterjee N Mackey S. Viewing pictures of a romantic partner reduces experimental pain: involvement of neural reward systems. PLoS One. (2010) 5:e13309. doi: 10.1371/journal.pone.0013309, PMID: 20967200 PMC2954158

[62] Mulej Bratec S Bertram T Starke G Brandl F Xie X Sorg C. Your presence soothes me: a neural process model of aversive emotion regulation via social buffering. Soc Cogn Affect Neur. (2020) 15:561–70. doi: 10.1093/scan/nsaa068, PMID: 32415970 PMC7328019

[63] Eippert F Finsterbusch J Bingel U Büchel C. Direct evidence for spinal cord involvement in placebo analgesia. Science. (2009) 326:404–4. doi: 10.1126/science.1180142, PMID: 19833962

[64] Rainville P. Brain mechanisms of pain affect and pain modulation. Curr Opin Neurobiol. (2002) 12:195–204. doi: 10.1016/s0959-4388(02)00313-6 12015237

[65] Lieberman MD Eisenberger NI. “A pain by any other name (rejection, exclusion, ostracism) still hurts the same,” in Social Neuroscience: People Thinking about Thinking People. eds. Cacioppo J. T. Visser P. S. Pickett C. L. (The MIT Press) (2005). 167–87.

[66] Haroush K Williams ZM. Neuronal prediction of Opponent’s behavior during cooperative social interchange in primates. Cells. (2015) 160:1233–45. doi: 10.1016/j.cell.2015.01.045, PMID: 25728667 PMC4364450

[67] Behrens TEJ Hunt LT Woolrich MW Rushworth MFS. Associative learning of social value. Nature. (2008) 456:245–9. doi: 10.1038/nature07538, PMID: 19005555 PMC2605577

[68] Tomlin D Kayali MA King-Casas B Anen C Camerer CF Quartz SR. Agent-specific responses in the cingulate cortex during economic exchanges. Science. (2006) 312:1047–50. doi: 10.1126/science.1125596, PMID: 16709783

[69] Sallet J Mars RB Noonan MP Andersson JL O’Reilly JX Jbabdi S. Social network size affects neural circuits in macaques. Science. (2011) 334:697–700. doi: 10.1126/science.1210027, PMID: 22053054

[70] Balliet D Lange PAMV. Trust, conflict, and cooperation: a meta-analysis. Psychol Bull. (2013) 139:1090–112. doi: 10.1037/a0030939 23231532

[71] Mortenson ST. Interpersonal trust and social skill in seeking social support among Chinese and Americans. Commun Res. (2009) 36:32–53. doi: 10.1177/0093650208326460

[72] Feeney BC Collins NL. A new look at social support. Personal Soc Psychol Rev. (2015) 19:113–47. doi: 10.1177/1088868314544222, PMID: 25125368 PMC5480897

[73] Barker C Pistrang N. Psychotherapy and social support. Clin Psychol Rev. (2002) 22:361–79. doi: 10.1016/s0272-7358(01)00101-5 17201191

[74] Grecucci A Frederickson J Job R. Editorial: advances in emotion regulation: from neuroscience to psychotherapy. Front Psychol. (2017) 8:985. doi: 10.3389/fpsyg.2017.00985, PMID: 28680409 PMC5479113

[75] DeRubeis RJ Siegle GJ Hollon SD. Cognitive therapy versus medication for depression: treatment outcomes and neural mechanisms. Nat Rev Neurosci. (2008) 9:788–96. doi: 10.1038/nrn2345, PMID: 18784657 PMC2748674

[76] Grecucci A Chiffi D Marzio FD Job R Frederickson J. “Anxiety and its Regulation: Neural Mechanisms and Regulation Techniques According to the Experiential-Dynamic Approach,” in New Developments in Anxiety Disorders. eds. Durbano F. Marchesi B. (IntechOpen), (2016).

[77] Sloan E Hall K Moulding R Bryce S Mildred H Staiger PK. Emotion regulation as a transdiagnostic treatment construct across anxiety, depression, substance, eating and borderline personality disorders: a systematic review. Clin Psychol Rev. (2017) 57:141–63. doi: 10.1016/j.cpr.2017.09.002, PMID: 28941927

[78] Zhao X-H Wang P-J Li C-B Hu Z-H Xi Q Wu W-Y. Altered default mode network activity in patient with anxiety disorders: an fMRI study. Eur J Radiol. (2007) 63:373–8. doi: 10.1016/j.ejrad.2007.02.006 17400412

[79] Messina I Bianco F Cusinato M Calvo V Sambin M. Abnormal default system functioning in depression: implications for emotion regulation. Front Psychol. (2016) 7:858. doi: 10.3389/fpsyg.2016.00858, PMID: 27375536 PMC4901076

[80] Sauer-Zavala S Boswell JF Gallagher MW Bentley KH Ametaj A Barlow DH. The role of negative affectivity and negative reactivity to emotions in predicting outcomes in the unified protocol for the transdiagnostic treatment of emotional disorders. Behav Res Ther. (2012) 50:551–7. doi: 10.1016/j.brat.2012.05.005, PMID: 22738907 PMC3408841

[81] Durnez J Moerkerke B Nichols TE. Post-hoc power estimation for topological inference in fMRI. NeuroImage. (2014) 84:45–64. doi: 10.1016/j.neuroimage.2013.07.072, PMID: 23927901

[82] Cremers HR Wager TD Yarkoni T. The relation between statistical power and inference in fMRI. PLoS One. (2017) 12:e0184923. doi: 10.1371/journal.pone.0184923, PMID: 29155843 PMC5695788

