# Supplementary material for: Social reappraisal of emotions is linked with the social presence effect in the default mode network
Source: Front Psychiatry. 2023 Mar 23;14:1128916. doi: 10.3389/fpsyt.2023.1128916 (PMC10076786; doi:10.3389/fpsyt.2023.1128916)
Supplement: Supplementary file 1 [file Data_Sheet_1.PDF]

Supplementary Material

Social Reappraisal of Emotions is Linked with the Social Presence Effect in the Default Mode Network

Xiyao Xie, Teresa Bertram, Saša Zorjan, Marina Horvat, Christian Sorg, Satja Mulej Bratec*

*** Correspondence:** satja.mulej@um.si

1 Supplementary Data

1.1 Aversive Pictures

80 aversive pictures with both high arousal and negative valence ratings were selected from the International Affective Picture System (IAPS) (Lang et al., 2008) and divided into 2 sets of 40 pictures, used in a counterbalanced way in the social reappraisal and social no-reappraisal conditions. Participants were thus exposed to different pictures in each condition to avoid confounding effects of repetition. To keep the design as balanced as possible, the two sets of pictures were comparable in arousal (p = 0.76) and valence (p = 0.97).

IAPS picture set 1 contained the following pictures: 9185, 2981, 3053, 6313, 9630, 3530, 9620, 3180, 9301, 1111, 6370, 3015, 3140, 2800, 3069, 9050, 9904, 2710, 9500, 3101, 3181, 3220, 9909, 6243, 3195, 9325, 9184, 9163, 3001, 3120, 9425, 9810, 3400, 2661, 3103, 3062, 9920, 2141, 3059, 3063. Mean valence of set 1 was 2.22 (SD = 0.52), while mean arousal was 6.06 (SD = 0.54).

IAPS picture set 2 contained the following pictures: 3110, 9921, 3150, 9423, 3060, 9414, 3068, 9412, 9008, 3191, 4664.2, 9435, 6350, 9623, 3213, 3230, 6520, 9295, 2375.1, 8230, 9910, 9901, 9321, 3019, 9253, 9040, 9187, 3225, 9599, 9600, 3131, 9611, 3130, 9410, 9905, 3215, 9140, 6022, 6231, 2205. Mean valence of set 2 was 2.26 (SD = 0.49), while mean arousal was 6.07 (SD = 0.71).

1.2 Psychotherapist’s sentences

**Social reappraisal sentences**	***English translation***
Sie wissen, Sie sind nicht betroffen.	*You know that you are not affected.*
Denken Sie daran, dass Sie hier sicher sind.	*Keep in mind that you are safe here.*
Vergessen Sie nicht, die Bilder haben nichts mit Ihnen zu tun.	*Don't forget, the pictures are not related to you.*
Vergessen Sie nicht, Sie sind nicht auf den Bildern dargestellt.	*Don't forget, you are not depicted in the pictures.*
Das hier ist eine Untersuchung, nicht die Realität.	*This is just an experiment, not reality.*
Denken Sie daran, dass die Bilder nur gestellt sind.	*You know that the scenes are just staged.*
Sie wissen, die Bilder betreffen Sie nicht.	*You know that these pictures do not affect you.*
Weder Sie noch Ihre Familienangehörigen sind betroffen.	*Neither you nor your relatives are involved.*
Sorgen Sie sich nicht, Sie sind hier sicher.	*Don't worry, you are safe here.*
Sie müssen keine Angst haben, die Bilder sind gestellt.	*You don’t have to be afraid, the pictures are staged.*
Erschrecken Sie nicht, die Bilder sind nur gestellt.	*Don't be scared, the pictures are just staged.*
Sie sind in Sicherheit, Ihnen kann nichts passieren.	*You are safe, nothing bad can happen to you.*
Auf den Bildern sind Szenen dargestellt.	*The pictures depict scenes.*
Die Bilder haben nichts mit ihrem Leben zu tun.	*The pictures don’t concern your life.*
Sie und Ihre Familie sind in Sicherheit.	*You and your family are safe.*
Sie wissen, dass die Bilder nichts mit der Realität zu tun haben.	*You know that the pictures are not related to reality.*
Sie wissen, dass Sie an einer Untersuchung teilnehmen.	*You know that you are participating in an experiment.*
Sie wissen, die Bilder sind nicht real.	*You know that the pictures are not real.*

Sie wissen, dass Sie hier sicher sind.	*You know that you are safe here.*
Atmen Sie ruhig weiter, Sie sind hier sicher.	*Keep breathing calmly, you are safe here.*
Denken Sie daran, die Bilder haben nichts mit Ihrem Alltag zu tun.	*Keep in mind that the pictures are not related to your everyday life.*
Denken Sie daran, die Bilder haben nichts mit Ihrer Situation zu tun.	*Keep in mind that the pictures have nothing to do with your situation.*
**Social no-reappraisal sentences**	***English translation***
Schauen Sie das Bild einfach an.	*Simply look at the picture.*
Schauen Sie das Bild an.	*Look at the picture.*
Verdeutlichen Sie sich den Inhalt des Bildes.	*Pay attention to the picture's content.*
Machen Sie sich den Inhalt des Bildes klar.	*Bring the content of the picture to your mind.*
Lassen Sie Ihre Gefühle zu.	*Experience your feelings.*
Lassen Sie Ihre Gefühle beim Betrachten des Bildes zu.	*Experience your feelings when looking at the picture.*
Ändern Sie Ihre Gefühle nicht.	*Don't change your feelings.*
Lassen Sie die Gefühle, die das Bild auslöst, zu.	*Experience the feelings induced by the picture.*
Schauen Sie sich das Bild an.	*Look at the picture.*
Machen Sie sich klar, was auf dem Bild dargestellt ist.	*Make what is shown on the picture clear to you.*
Seien Sie sich im Klaren über die Bedeutung des Bildes.	*Be aware of the picture's meaning.*

2 Supplementary Figures

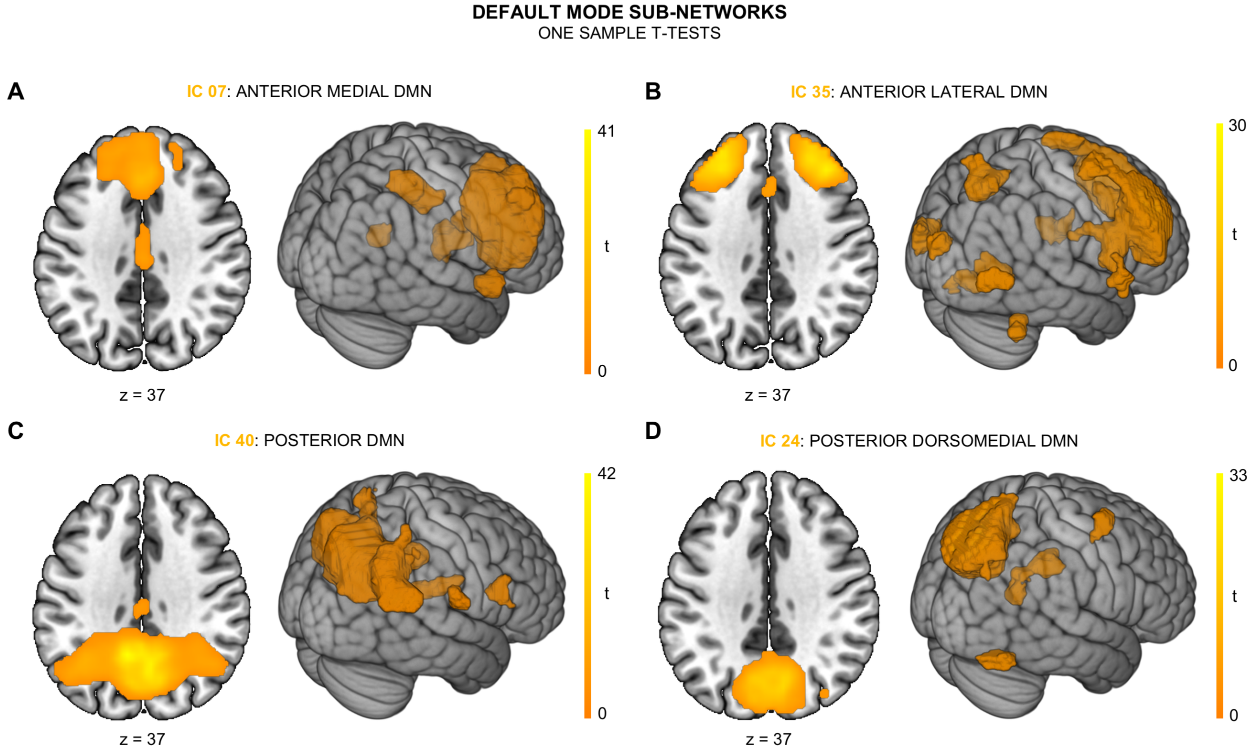

Supplementary Figure 1. Default mode network (DMN) sub-networks. The 4 networks were chosen based on existing templates of 4 intrinsic connectivity networks representing DMN sub-networks. **(A)** Independent component (IC) 7 was chosen as the anterior medial DMN; **(B)** IC 35 was the anterior lateral DMN; **(C)** IC 40 was the posterior DMN; **(D)** IC 24 coincided most closely with the posterior dorsomedial DMN.

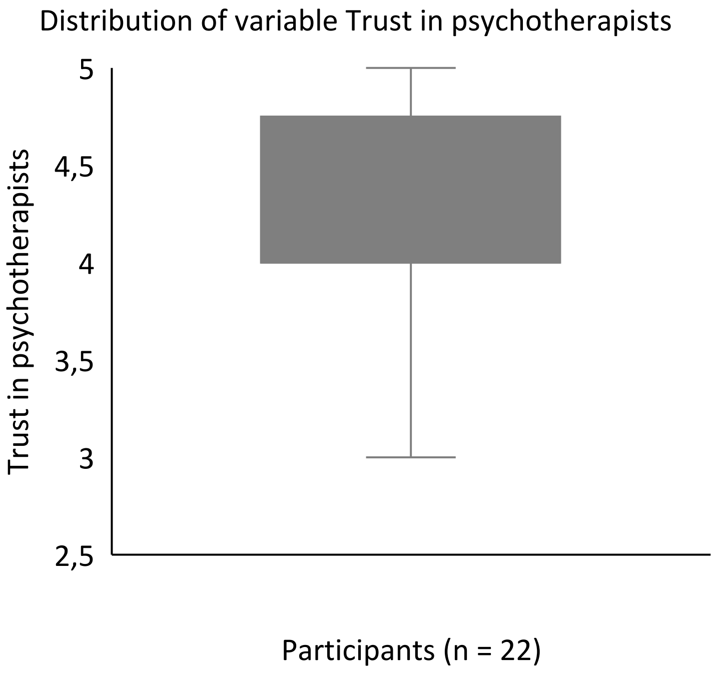

Supplementary Figure 2. A box and whisker plot, showing the distribution of values for the variable Trust in psychotherapists. The range of participants' trust scores was 3 – 5, mean = 4.35, SD = 0.53.

3 Supplementary Tables

Supplementary Table 1. Supportive social presence-related change in iFC within the aDMN.

Region	Cluster size	Peak MNI coordinates x	y	z	Peak z	p (FWE-corrected)
**Social > Non-Social**						
Anterior Cingulate Gyrus, Paracingulate Gyrus	48	-3	23	37	3.99	0.046
**Non Social > Social**						
No significant clusters.						

Note. Based on a paired *t*-test social > non-social during resting state, FWE-corrected ($p < 0.05$) at the cluster level, within the aDMN network, based on a height threshold of $p < 0.005$ (i.e., extent threshold of 47 voxels). Anatomical regions were identified with the SPM Anatomy Toolbox (Eickhoff et al., 2005); shown are the top two regions for each cluster (assignment based on maximum probability).

Supplementary Table 2. Social Reappraisal Network.

Region	Cluster size	Peak MNI coordinates x	y	z	Peak z	p (FWE-corrected)
**IAPS Picture Presentation: Social Reappraisal > Social No-Reappraisal**						
R Angular gyrus, Lateral Occipital Cortex	323	51	-58	37	6.31	0.002
L Lateral Occipital Cortex, Angular Gyrus	542	-48	-61	46	5.81	0.000
R & L Frontal Pole, Superior Frontal Gyrus	2430	45	29	40	5.57	0.000
Precuneus Cortex, Cingulate Gyrus (posterior)	539	-3	-70	43	5.24	0.000
L Cerebellum Crus II, Cerebellum Crus I	225	-36	-61	-41	4.83	0.016

Note. Based on a paired *t*-test social reappraisal > social no-reappraisal for the time window of negative IAPS picture presentation, whole-brain FWE-corrected ($p < 0.05$) at the cluster level, based on a height threshold of $p < 0.005$ (i.e., extent threshold of 174 voxels). Anatomical regions were identified with the SPM Anatomy Toolbox (Eickhoff et al., 2005); shown are the top two regions for each cluster (assignment based on maximum probability).
